# Determining the Efficacy of Electronic Cognitive Behavioral Therapy for Generalized Anxiety Disorder Compared to Pharmaceutical Interventions: Protocol for a Quasi-Experimental Study

**DOI:** 10.2196/27772

**Published:** 2021-05-27

**Authors:** Nazanin Alavi, Callum Stephenson, Megan Yang, Amirhossein Shirazi, Yijia Shao, Anchan Kumar, Caitlin S Yee, Shadé Miller, Anthi Stefatos, Maedeh Gholamzadehmir, Zara Abbaspour, Archana Patel, Charmy Patel, Taras Reshetukha, Mohsen Omrani, Dianne Groll

**Affiliations:** 1 Department of Psychiatry Queen's University Kingston, ON Canada; 2 Centre for Neuroscience Studies Queen's University Kingston, ON Canada; 3 OPTT Inc Toronto, ON Canada; 4 Department of Psychology University of Toronto Toronto, ON Canada

**Keywords:** eHealth, mental health, anxiety, generalized anxiety disorder, cognitive behavioral therapy, psychotherapy, online, internet, electronic, virtual, mental health care

## Abstract

**Background:**

Generalized anxiety disorder (GAD) is an extremely prevalent and debilitating mental health disorder. Currently, the gold standard treatment for GAD is cognitive behavioral therapy (CBT) and/or pharmacotherapy. The most common medications used to treat GAD are selective serotonin reuptake inhibitors and selective norepinephrine reuptake inhibitors. While CBT is the gold standard treatment for GAD, it is costly, time-consuming, and often inaccessible. Fortunately, the electronic delivery of CBT (e-CBT) has emerged as a promising solution to address these barriers. e-CBT has shown to offer comparable results to in-person CBT while improving accessibility for patients and time efficiency for clinicians.

**Objective:**

This study aims to investigate the treatment efficacy of e-CBT compared to and in conjunction with pharmacotherapy for GAD.

**Methods:**

This study will use a quasi-experimental design to allow patients the freedom to choose which treatment modality they would like to receive. Participants with a diagnosis of GAD will be enrolled in 1 of 3 possible treatment arms: (1) e-CBT, (2) medication, or (3) a combination of e-CBT and medication. The e-CBT program will include a 12-week psychotherapy program delivered through the Online Psychotherapy Tool—a secure, cloud-based, digital mental health platform. The treatment efficacy of e-CBT will be compared with that of medication alone and medication in combination with e-CBT.

**Results:**

The study received ethics approval in April 2019 and participant recruitment began in June 2019. Participant recruitment has been conducted through social media advertisements, physical advertisements, and physician referrals. To date, 146 participants (e-CBT: n=53; medication: n=49; combination: n=44) have been recruited. Data collection is expected to conclude by June 2021, and data analysis is expected to be completed by October 2021. Linear regression (for continuous outcomes) and binomial regression (for categorical outcomes) analysis will be conducted using interpretive qualitative methods.

**Conclusions:**

If either the efficacy of e-CBT is shown to be comparable to that of medication or the effects of both treatments are augmented when used in tandem, these findings could have major implications on the mental health care system. e-CBT is a more accessible and affordable treatment that could increase mental health care capacity 4-fold if proven viable.

**Trial Registration:**

ClinicalTrials.gov NCT04478526; https://clinicaltrials.gov/ct2/show/NCT04478526

**International Registered Report Identifier (IRRID):**

DERR1-10.2196/27772

## Introduction

### Background and Rationale

An estimated 450 million people suffer from mental and/or behavioral disorders globally [1]. Anxiety disorders are some of the most common mental health disorders, with generalized anxiety disorder (GAD) being the second most prevalent among them [2,3]. As the demand for treatment increases, mental health care systems globally are becoming overwhelmed and in need of accessible, cost-effective, time-friendly solutions [4].

Currently, the gold standard treatments for GAD are pharmacotherapy and/or psychotherapy [5]. The recommended first-line pharmacotherapies are selective serotonin reuptake inhibitors (SSRIs) and selective norepinephrine reuptake inhibitors (SNRIs) [5]. Regarding psychotherapy, 12-20 sessions of individual cognitive behavioral therapy (CBT) is the first-line treatment [5]. Individual CBT is effective at improving patient quality of life and decreasing psychological distress in times of crisis, with long-term effects being seen [5-9].

While these pharmacotherapies and CBT are generally effective, neither are without their drawbacks. In terms of pharmacotherapy, a large stigma, potential adverse effects, polypharmacy, and personal reasons deter many patients from opting for this treatment. Regarding individual CBT, high costs, long waiting lists, privacy concerns, and treatment times only available during regular work hours often make it inaccessible. Due to these limitations, there is an urgent need to create a treatment modality for GAD that can be accessible and cost-effective without sacrificing the quality of care and treatment efficacy.

The delivery of CBT asynchronously through the internet (e-CBT) appears to be a viable solution to address the limitations of CBT. e-CBT has been proven to be clinically efficacious, increase treatment adherence, yield high treatment satisfaction, and offer comparable results to in-person CBT [10-17]. Given the structured nature of CBT, predesigned content can be provided to patients, allowing them to access it anywhere at any time, saving health care providers time and costs, while increasing care capacity. While e-CBT has been shown in the literature to be effective in treating anxiety symptoms, the method of delivery has largely been through self-help, with patient-therapist interaction occurring through email [11-15]. While self-help programs can be beneficial, therapist-patient engagement is associated with increases in treatment effectiveness [18-21]. Moreover, email as the primary form of communication between therapist and patient is both insecure and nonscalable. Therefore, a secure, scalable, and therapist-guided delivery of e-CBT is needed. While using a different platform still requires clinicians to provide individual feedback (similar to email), the scalability comes from the overall clinic-like management and automatic distribution of materials to patients following the completion of their assignments.

While it is known that e-CBT offers comparable results to in-person CBT when treating GAD, it has yet to be investigated whether a combination therapy of e-CBT and pharmacotherapy offers an augmented benefit to the individual. Additionally, an investigation of the efficacy of pharmacotherapy versus e-CBT through a secure platform has not been conducted to date. The potential of discovering a new gold-standard combination therapy for the treatment of GAD could have significant implications in the health care field, with greater accessibility for patients and time-efficiency for health care providers.

### Objectives

In this study, a secure and scalable e-CBT program will be delivered to individuals with GAD. This program will be delivered through the Online Psychotherapy Tool (OPTT), which is a secure, cloud-based platform designed specifically for the online delivery of psychotherapy [10,22-27]. There will be 12 e-CBT modules that mirror in-person, individual CBT content. Participants will be offered either e-CBT, medication, or a combination of e-CBT and medication. The following project aims to investigate the efficacy of e-CBT in the treatment of GAD compared to and in conjunction with current pharmacotherapy strategies. By using OPTT, we hypothesize that this psychotherapy intervention will improve patient quality of life and decrease symptom severity in individuals with GAD independent of medication use.

## Methods

### Study Design

This study will use a quasi-experimental design to allow participants the freedom to choose which treatment they would like to receive. This research design aims to be naturalistic by mimicking the decisions made by patients and physicians regarding their autonomy to choose a course of treatment. The treatments provided within the study also aim to replicate evidence-based best practice clinical guidelines for the treatment of GAD. All procedures have been approved by the Queen’s University Health Sciences and Affiliated Teaching Hospitals Research Ethics Board (HSREB) (Multimedia Appendix 1, Multimedia Appendix 2).

### Participants

A total of 165 patients (e-CBT: n=55; medication: n=55; combination: n=55) aged 18-65 years will be recruited at Queen’s University from outpatient psychiatry clinics at Kingston Health Sciences Centre sites (Hotel Dieu Hospital and Kingston General Hospital), Providence Care Hospital, family doctors, physicians, clinicians, and self-referrals in Kingston, Ontario, Canada. Interested participants will meet with a research coordinator who will provide them with a study letter of information that they will read before obtaining informed consent. Additionally, it will be explained to the participants that they will not always have access to their therapist and that the program is not to be used as a crisis resource. Once informed consent is obtained, a psychiatrist on the research team will evaluate the participants through secure video appointments. During these appointments, a diagnosis of GAD will be confirmed using the Diagnostic and Statistical Manual of Mental Disorders, Fifth Edition (DSM-5) [28].

Inclusion criteria include being between 18 and 65 years of age at the start of the study, a diagnosis of GAD according to the DSM-5, the competence to consent to participate, the ability to speak and read English, and consistent and reliable access to the internet. Exclusion criteria include active psychosis, acute mania, severe alcohol or substance use disorder, and/or active suicidal or homicidal ideation. Additionally, participants will be excluded if they are receiving another form of psychotherapy, as this could have a confounding effect on the efficacy of treatment. Participation in the study will be discontinued if the participant is noncompliant with their treatment program. Regarding medication, noncompliance will be defined as stopping the medication altogether or skipping more than 3 days of doses in a row. With e-CBT treatment, noncompliance will be defined as missing more than 2 weeks of e-CBT sessions. If a participant is deemed to be in an acute crisis by self-report or by the psychiatrist in charge of their care, their treatment will be halted and they will be directed to the proper resources (eg, emergency department, crisis lines, etc). If deemed eligible for the study, participants will be presented with all 3 arms of the study by the psychiatrist, who will discuss the recommended treatment plan. In collaboration with the psychiatrist, the participant will decide which of the treatment arms they would like to take part in.

### Procedures

At baseline, participants successfully enrolled in the study will complete a demographic questionnaire and the following clinically validated symptomatology questionnaires: the Quality of Life Enjoyment and Satisfaction Questionnaire–Short Form (Q-LES-Q-SF), the 7-item Generalized Anxiety Disorder Questionnaire (GAD-7), and the 42-item Depression Anxiety Stress Scale (DASS-42). All participants will complete the questionnaires biweekly (at weeks 0, 2, 4, 6, 8, 10, and 12) for the duration of treatment and at a 6-month follow-up. Participants in the e-CBT or combination arms will complete these questionnaires directly through OPTT. For participants in the medication arm, the questionnaires will be completed during their appointments. Once the treatment has been decided upon by the participant in collaboration with the psychiatrist, treatment will commence according to the arms explained below. Participants in the combination arm will begin the e-CBT program and pharmacotherapy simultaneously.

### e-CBT Protocol

All e-CBT modules are designed to mirror standard in-person CBT for GAD. The basis of the therapy is to help participants understand the interconnectivity of their thoughts, behaviors, emotions, physical reactions, and environment. This is achieved through presenting information, learning coping skills, and practicing these skills through homework. These coping skills will include actions such as deep breathing and meditating, goal setting, thought recording, and activity schedules. Through the course of this program, participants will work on refocusing their beliefs and thoughts to more realistic states in which they can better cope with their anxiety. By improving thought processes, participants will have the ability to better react to events in their environment and develop coping strategies.

Participants will complete approximately 30 e-CBT slides each week through the OPTT platform. Each session is expected to last approximately 50 minutes. The slides will highlight different topics each week and include general information, an overview of skills, and homework. The homework included in each session will be submitted through OPTT and reviewed by a therapist assigned to the participant. Therapists will provide personalized feedback every week to their participants within 3 days of submission. Participants will have access to these online sessions at any point throughout the week and can complete them in multiple blocks or all at once. Weekly homework submission for feedback will be mandatory before being eligible for the next session. Feedback will be reviewed by one of the psychiatrists on the team before submission to the participants.

### Therapist Training

All therapists on the research team have experience in psychotherapy delivery and are trained by a psychiatrist involved in the project. Additionally, all therapists learn the specifics of the modules covered in treatment, along with the standard care pathway. The therapists are a combination of medical graduates and residents, graduate students, and trained research assistants. Before working with any patients, therapists will provide practice feedback on simulated sessions that will be analyzed by the psychiatrists on the team to ensure that the quality of care is adequate. All therapists will be supervised by the lead psychiatrist on the research team who is an expert in online psychotherapy delivery. Moreover, homework feedback is only sent to the patient after it is read, edited, and approved by the supervisor. Any issues regarding OPTT are handled through OPTT technical support, which can be accessed at any time.

### Medication Protocol

**Figure 1:**
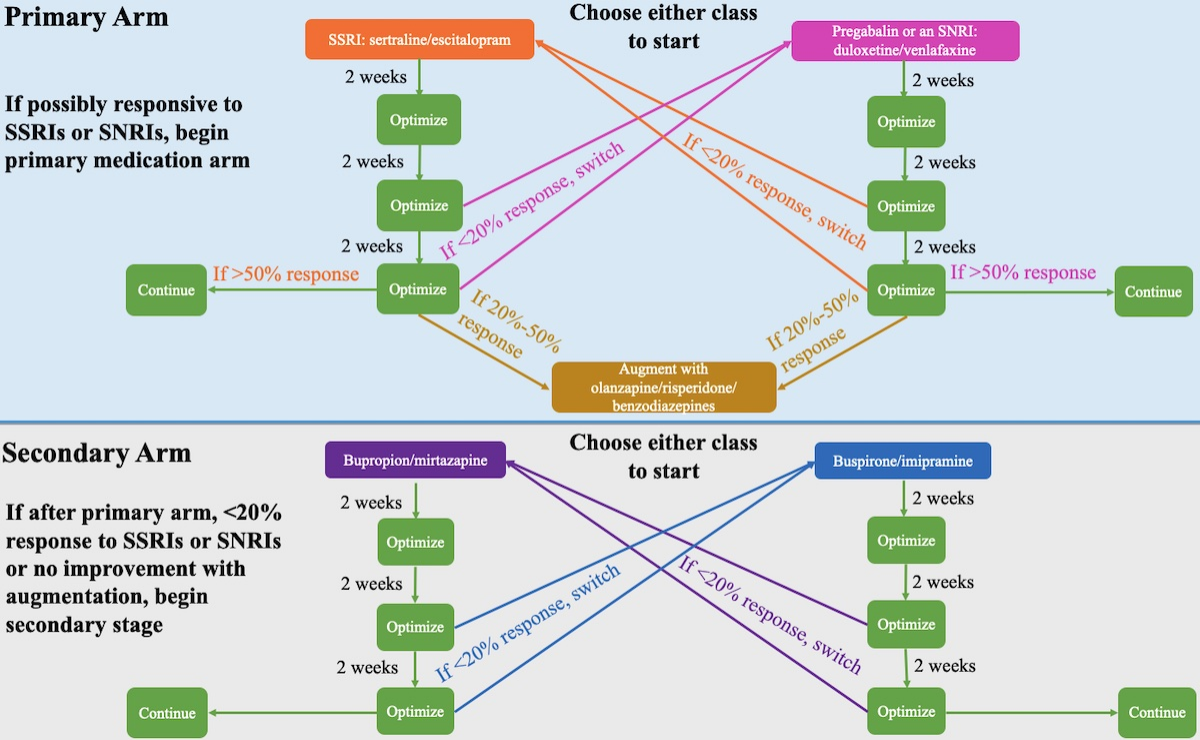
Flowchart of the 12-week generalized anxiety disorder pharmacotherapy protocol. SNRI: selective norepinephrine reuptake inhibitor; SSRI: selective serotonin reuptake inhibitor.

Participants in the medication or combination arm will attend biweekly medication reconciliation appointments with their psychiatrist at the clinic. During the intake appointment, participant medication history (including any current medications) will be collected. If a participant is taking medication for GAD that is not one of the recommended ones in the protocol, they will be switched to a suggested drug. It is required that a participant's medication remain unchanged for 6 weeks before the start of the study and during the study. The psychiatrist will suggest medications according to Canada’s best practice guidelines for the treatment of GAD. The pharmacotherapy protocol is summarized in Figure 1.

If a participant has never taken an SSRI or SNRI, they will commence the primary medication arm. The 2 classes of medications within the primary arm will be described to the participant, and with the recommendation of the prescribing psychiatrist the participant will begin either an SSRI (sertraline or escitalopram) or pregabalin/SNRI (duloxetine or venlafaxine). If the participant has previously been deemed unresponsive to either an SSRI or an SNRI/pregabalin, they will commence the primary medication arm. The participant will start the medication class that they have not been previously deemed unresponsive to (eg, if previously unresponsive to sertraline, the participant will commence the SNRI class). Previous unresponsiveness will be defined as anxiety symptoms not improving after treatment with the maximum tolerated dose of the specific medication for a duration of 8 weeks. If a participant is deemed unresponsive to both an SSRI and an SNRI/pregabalin, they will commence the secondary medication arm. The 2 classes of medications within the second arm will be described to the participant and, with the recommendation of the prescribing psychiatrist, they will begin either bupropion/mirtazapine or buspirone/imipramine.

At the participant’s second appointment (2 weeks on the medication), their medication will be maintained and optimized, regardless of whether a response is reported. At the third appointment (4 weeks on the medication), the medication will be optimized if a partial response is reported or switched according to the “medication switch protocol” if no response is reported. Partial response will be defined as an improvement of ≥20% in GAD-7 score compared with baseline. If the medication is switched (<20% improvement in GAD-7 score compared with baseline), the 6-week protocol will recommence with the new medication. At the fourth appointment (6 weeks on the medication), the dosage will be optimized if the participant is responding well to the medication and reports an improvement in GAD-7 score of >50% if within the primary medication arm or ≥20% if within the secondary medication arm compared with baseline. If this is the case, the participant will remain on that medication for the remainder of the 12-week study. If the participant does not present an improvement of >20% in their GAD-7 score compared with baseline, the medication will be switched according to the “medication switch protocol” and the 6-week protocol will recommence. If the participant is in the primary medication arm and reports a 20% to 50% improvement in GAD-7 score compared with baseline after 6 weeks on the new medication, the medication will be augmented with either olanzapine, risperidone, or benzodiazepines. Benzodiazepines have shown efficacy as adjunctive therapy in the treatment of anxiety; they have especially been helpful with decreasing level of agitation. Adjunctive olanzapine and risperidone demonstrated efficacy in patients who remained symptomatic after 6 weeks of antidepressant therapy [29].

### Medication Switch Protocol

If a participant is unresponsive to medication after 4 or 6 weeks of administration (<20% improvement in GAD-7 score compared with baseline), their medication will be switched to another class. If the participant has a history of nonresponse to any of the 4 medication classes, these classes will be removed as treatment options. If a participant started in the primary or secondary medication arm and has not previously demonstrated nonresponse to the second class of medications within that arm, they will be switched to the second class of pharmaceuticals within that arm. If a participant started in the primary arm and was previously unresponsive to the second class of pharmaceuticals within that arm, they will begin the secondary medication arm if necessary.

### Ethics and Data Privacy

All procedures have been approved by the Queen’s University HSREB. For privacy purposes, participants are only identifiable by an ID number on the platform and hard copies of the consent forms with participants’ identities are stored securely on-site and will be destroyed 5 years after study completion. Participant data are only accessible by the care providers directly assigned to that participant and only anonymized data are provided to the analysis team members. Participants have the option to withdraw from the study at any point and request for their data to be removed from the analysis. However, since the collected data are considered a medical record, they will not be permanently deleted for 10 years after treatment.

The online platform used for the study (ie, OPTT) is compliant with the Health Insurance Portability and Accountability Act, Personal Information Protection and Electronic Documents Act, and Service Organization Control 2. Additionally, all servers and databases are hosted in Amazon Web Services Canada's cloud infrastructure, which is managed by MedStack to assure that all provincial and federal privacy and security regulations are met. OPTT does not collect any identifiable personal information or internet protocol addresses for privacy purposes. OPTT only collects anonymized metadata to improve its service quality and provide advanced analytics to the clinician team. OPTT encrypts all data, and no employee has direct access to participant data. All encrypted backups are kept in the S3 storage that is dedicated to Queen’s University, located in Kingston, Ontario, Canada.

### Data Analysis

Initially, all data will be examined for missing, nonsensical, and outlying variables. Missing data will be treated as missing and not imputed (ie, analyzed on a per-protocol basis). The participant population of this study was intentionally oversampled to account for dropouts/withdrawals. Based on previous research, an anticipated dropout rate of up to 30% was factored in. Using the GAD-7 score as the primary outcome, a 30% change is considered clinically significant. Therefore, a sample size of 55 participants in each arm of the study would be sufficient for detecting significant results with *P*=.05 and a power of 0.95. Data collection will occur biweekly and at a 6-month follow-up. Using Mann-Whitney U tests, demographic information can be compared between participants who complete the program and those who withdraw prematurely in the hopes of identifying possible differences between the 2 groups. Moreover, an intention-to-treat analysis will be conducted to evaluate the clinical effects of treatment on participants who withdraw prematurely. Linear regression analysis (for continuous outcomes) and binomial regression analysis (for categorical outcomes) will be used to identify variables associated with the outcome measures (GAD-7, DASS-42, and Q-LES-Q-SF). This will occur over the 4 measurement time points while controlling for demographic variables, including age and gender. Additionally, differences between study arms will be analyzed.

Other quantitative measures for the e-CBT group will be gathered by extrapolating recorded information directly through the OPTT application (ie, the number of logins per day, the amount of time spent logged in, etc). Qualitative measurement analyses will be done to inquire about the role of personal, social, and cultural factors in enabling or constraining the use of e-CBT. Findings will identify factors related to the utility, feasibility, and accessibility of e-CBT from the perspectives of users and providers. Interpretive qualitative methods are ideal for gathering in-depth descriptions of user experience and meaning.

## Results

The study received ethics approval from the Queen’s University HSREB in April 2019, and the recruitment of participants began in June 2019. Participant recruitment has been conducted through social media advertisements, physical advertisements, and physician referrals. To date, 146 participants have been recruited (e-CBT: n=53; medication: n=49; combination: n=44). Data collection is expected to conclude by June 2021, and data analysis is expected to be completed by October 2021. Linear regression will be used to analyze continuous outcomes, and binomial regression will be used to analyze categorical outcomes.

## Discussion

GAD is a prevalent mental illness that is overwhelming the mental health care system. Innovative, efficacious, and accessible treatments are needed to address the issues with current treatment options. Developing an online psychotherapy clinic with predesigned therapy modules can drastically increase care capacity without sacrificing the quality of care. Investigating the effectiveness of an e-CBT program compared to pharmacotherapy and a combination of the 2 can provide valuable insight into new treatment developments. Outcomes of this study will be shared as a preprint on bioRxiv.org for the rapid dissemination of findings. We will also hold multiple online workshops for other clinicians interested in implementing this approach and provide technical and academic support to deploy this solution in their respective practices. This will ensure that the findings can be efficiently incorporated into clinical practice. If feasible, an online psychotherapy clinic can provide significant time and financial savings to the health care system while providing an equitable and accessible method of treatment delivery for patients.

## Appendix

Multimedia Appendix 1 GUIDED report checklist.

Multimedia Appendix 2 TIDieR report checklist.

## Abbreviations

CBT: cognitive behavioral therapy
DASS-42: 42-item Depression Anxiety Stress Scale
DSM-5: Diagnostic and Statistical Manual of Mental Disorders, 5th Edition
e-CBT: electronically delivered cognitive behavioral therapy
GAD: generalized anxiety disorder
GAD-7: 7-item Generalized Anxiety Disorder Questionnaire
HSREB: Health Sciences and Affiliated Teaching Hospitals Research Ethics Board
OPTT: Online Psychotherapy Tool
Q-LES-Q-SF: Quality of Life Enjoyment and Satisfaction Questionnaire–Short Form
SNRI: selective norepinephrine reuptake inhibitor
SSRI: selective serotonin reuptake inhibitor
